# More Attacks and Analgesic Use in Old Age: Self-Reported Headache Across the Lifespan in a German Sample

**DOI:** 10.3389/fneur.2019.01000

**Published:** 2019-10-17

**Authors:** Britta Müller, Thomas Dresler, Charly Gaul, Änne Glass, Tim P. Jürgens, Peter Kropp, Ruth Ruscheweyh, Andreas Straube, Stefanie Förderreuther

**Affiliations:** ^1^Institute of Medical Psychology and Medical Sociology, University Medicine Rostock, Rostock, Germany; ^2^Department of Psychiatry and Psychotherapy, University Hospital Tübingen, Tübingen, Germany; ^3^LEAD Graduate School & Research Network, University of Tübingen, Tübingen, Germany; ^4^Migraine and Headache Clinic Königstein, Königstein, Germany; ^5^Institute for Biostatistics and Informatics in Medicine and Ageing Research, University Medicine Rostock, Rostock, Germany; ^6^Department of Neurology, University Medicine Center Rostock, Rostock, Germany; ^7^Department of Neurology, Ludwig Maximilian University of Munich, Munich, Germany

**Keywords:** migraine, tension-type headache, lifespan, population-based study, 6-month prevalence, headache attacks, headache impact, use of analgesics

## Abstract

**Background:** Reliable population-based data on the prevalence and characteristics of primary headache across the lifespan are essential. However, robust data are lacking.

**Methods:** We utilized questionnaire data from a random general population sample in Germany, that comprised 2,478 participants aged ≥14 years. A standardized questionnaire addressing headache and headache treatment was filled in during the face-to-face survey.

**Results:** The 6-month prevalence of self-reported headache in the total sample amounted to 39.0% (known diagnosis of migraine 7.2%; tension-type headache 12.4%; another diagnosis or unknown diagnosis 23.4%). Age-specific prevalence rates were 37.9% (14–34 years), 44.6% (35–54 years), 38.5% (55–74 years), and 26.9% (≥75 years). Compared to age group 14–34, participants aged 35–54 were more (*OR* = 1.29, 95%-*CI* 1.05–1.60, *p* = 0.018) and those aged ≥75 were less (*OR* = 0.55, 95%-*CI* 0.40–0.76, *p* < 0.001) likely to have any headache. Of the participants with headache, 79.5% reported headache on <4 days per month, 15.6% on 4–14 days per month and 4.9% on >14 days per month. The frequency of headache did not differ significantly between age groups in men [$\chi_{\left(3,\;N\;=\;384\right)}^{2}$ = 1.45, *p* > 0.05], but in women [$\chi_{\left(3,\;N\;=\;651\right)}^{2}$ = 21.57, *p* < 0.001]: women aged ≥75 years were over-represented in the group reporting 4–14 headache days per month. The analgesic use (days per month) differed significantly between age groups among participants with headache on <4 days per month and on >14 days per month: 1.8 (14–34 years), 2.5 (35–54 years), 3.2 (55–74 years), and 3.4 (≥75 years), respectively 7.9 (14–34 years), 11.4 (35–54 years), 18.4 (55–74 years), and 22.8 (≥75 years).

**Conclusions:** In general, the prevalence of headache decreases with age. However, older women suffer from more frequent attacks and older participants take analgesics on more days per month than younger participants. This might put them at risk of medication overuse which may lead to medication overuse headache. More research is needed to understand these specifics in headache frequency and treatment behavior in older people.

## Introduction

Headache is the most frequent neurological disorder worldwide (1). According to estimates, the 1-year prevalence is around 38% for tension-type headache (TTH), and 10% for migraine (2, 3). These two types of headache account for 13% of all disability-adjusted life-years (DALYs) caused by neurological disorders (1). For purposes of healthcare planning, reliable population-based data on the characteristics of headache across the lifespan, such as prevalence, frequency of attacks and headache impact, are essential. Additional data on behavioral patterns across the lifespan in people with headache, such as physician consultation and use of medication, are also important. There are hardly any robust research results available.

Previous findings in prevalence refer almost exclusively to children and adolescents as well as young and middle-aged adults (4). Prevalence studies including older persons suffer from different limitations. First, a number of studies date back at least two decades (5–8). Second, some studies do not differentiate between different age groups among elderly people (6, 8–11). Third, many study samples make it difficult to generalize the findings to the whole population because they exclude either rural (12, 13) or urban residents (14–16). Age-specific patterns of attack frequency, medical use and headache impact have rarely been studied.

The aim of the present analysis is to (a) describe the 6-month prevalence of self-reported headache across the lifespan in general and (b) to identify age-specific characteristics of frequency of headache, headache impact and frequency of analgesic use within the German-speaking population aged ≥14 years.

## Methods

### Participants

The analysis is based on data from a random general population sample in Germany with participants aged 14–94 years. A cross-sectional questionnaire survey was performed through face-to-face contacts conducted by an independent demographic consulting company (USUMA, Berlin, Germany). The sociodemographic data was collected using a standardized face-to-face interview. For the collection of headache-related data we used standardized questionnaires filled in by the participants themselves. For sample selection, random multi-stage sampling procedures were employed. First, 258 regional sample points in Germany were determined (stage 1). Subsequently, 19 households per sample point were selected using the random-route procedure (stage 2) (17). Members of households, who met the inclusion criteria (age above 14, able to read, and understand German) were randomly selected using the Kish-selection-grid technique (stage 3) (18). The sample was designed to be representative in terms of age, sex, household size, and population by federal state. In total, 4,838 subjects were selected for the study, of whom 52% participated (*N* = 2,510). Reasons for non-participation were refusal to take part (*n* = 1,453), three unsuccessful attempts to contact the household member (*n* = 863), and illness of the selected subjects or his/her incapacity to follow the interview (*n* = 8). The final sample consisted of 2,510 interviewed participants. From the current analysis 32 subjects were excluded [reasons for exclusion were missing answers to following questions: (a) occurrence of headache in the last 6 months *n* = 5; (b) headache diagnosis *n* = 13; (c) headache frequency *n* = 15]. Thus, *n* = 2,478 interviewees were entered into the present analysis. A comparison of included subjects vs. drop-outs revealed no differences regarding sex composition, $\chi_{\left(1,\;N\;=\;2,510\right)}^{2}$ = 1.09, *p* = 0.373 and mean age, *U* = 35947.50; *p* = 0.364, *r* = 0.02. An adjustment factor was calculated on the basis of the German population structure regarding age, sex, household size, and population by federal state. German population parameters were provided by data from the 2016 Microcensus conducted by the German Federal Statistical Office. Based on the adjustment factor a weighted random sample was created, the structure of which corresponds to that of the German population (19). Data collection took place from September to November 2016. All participants gave their written informed consent. The Ethics Committee of the Faculty of Medicine, University of Leipzig, reviewed and approved the study (297/16-ek).

### Questionnaire

The standardized questionnaire about headache and headache treatment consisted of 36 items. The introductory screening question was “Did you have a headache in the last 6 months?” The advantage over lifetime prevalence is that data depicting headache in a maximum of the last 12 months show a higher reliability due to a lower recall bias, particularly among older subjects (4). The headache frequencies were assessed using a five-point ordinal scale: (1) <1 day per month; (2) 1–3 days per month; (3) 4–14 days per month; (4) >14 days per month, but not daily; (5) daily. For statistical analysis we summarized the five categories into three classes: <4 days per month, 4–14 days per month, >14 days per month. In those participants having indicated the presence of headache, data regarding headache types was assessed by self-reporting using an item allowing multiple answers. The question used here was: “Do you know the diagnosis of your headache?” Response options were “migraine,” “TTH,” “cluster headache,” “other headache,” and “unclear headache (unknown diagnosis).” We summarized the headache types “cluster headache,” “other headache,” and “unclear headache (unknown diagnosis)” to the category “other headache.” As a reliable distinction between migraine with and without aura cannot be made solely by such a questionnaire, it was not assessed in our study (4). The impact of headache on daily life was assessed using the German version of the Headache Impact Test (HIT-6) (20). The HIT-6 is a six-item, self-administered questionnaire with three items assessing the impact of headache during the past 4 weeks and three items without a specific timeframe. Five response categories were given: “never,” “rarely,” “sometimes,” “very often,” and “always.” The total score can range from 36 to 78. Higher scores indicate a greater impact of headache on the ability to function on the job, at school, at home and in social situations. The HIT-6 provides a grading indicating four levels of headache impact: little or no impact (<50), some impact (50–55), substantial impact (56–59), and severe impact (≥60). To determine lifetime physician consultation for headache, participants were asked if they ever consulted a physician (or more than one) because of their headaches (yes/no). Analgesic use was assessed with the question: “How many days per month do you use analgesics on average?” Additionally, we used a standardized sociodemographic questionnaire to assess following parameters: age, sex, school education, and living alone (yes/no). Data regarding the community size was taken from the sampling plan.

### Statistical Analysis

The data of the weighted random sample were analyzed using descriptive statistics. The 6-month prevalence of self-reported headache was estimated with 95% confidence interval (CI). To test for differences between sociodemographic characteristics and sex, we used Fisher‘s exact test, Welch's *t*-test and Pearson's chi-squared test. To identify the factors associated with headache, we conducted a multivariate binary logistic regression that included sex and age group. We conducted ANOVAs to assess the effects of sex and age group on frequency of headache, analgesic use and headache impact (21). Differences were considered significant if the two-tailed *p*-value was *p* < 0.05. To analyze categorical variables we calculated adjusted Pearson residuals (22). We tested these residuals in the cells using a Bonferroni correction. For 6-month prevalence of headache, scatterplot smoothing LOESS curves were fitted to both men and women, with non-linear regression. Statistical analyses were performed using IBM SPSS Statistics 23.

## Results

### Sociodemographic Characteristics

The sociodemographic characteristics of the weighted sample stratified for sex are presented in Table 1. The age of the participants ranged from 14 to 94 years.

**Table 1:** Sociodemographic characteristics of the study sample according to sex.

**Variable**		**Total sample**	**Men**	**Women**	***p-*Value**
		***n* = 2,477**	***n* = 1,215**	***n* = 1,262**	
Age, mean (*SD*)		48.71 (19.15)	47.47 (18.91)	49.90 (19.31)	0.002^a^
Age group, *n* (%)	14–24 years	319 (12.9)	166 (13.7)	153 (12.1)	0.059^b^
	25–34 years	359 (14.5)	196 (16.1)	163 (12.9)	
	35–44 years	360 (14.4)	172 (14.2)	188 (14.9)	
	45–54 years	449 (18.1)	225 (18.5)	224 (17.7)	
	55–64 years	388 (15.7)	191 (15.7)	197 (15.6)	
	65–74 years	327 (13.2)	143 (11.8)	184 (14.6)	
	75 years or older	275 (11.1)	122 (10.0)	153 (12.1)	
High school graduation (yes), *n* (%)		520 (21.1)	283 (23.3)	237 (18.9)	0.008^c^
Marital status, *n* (%)	Unmarried	721 (29.2)	407 (33.6)	314 (25.0)	<0.001^b^
	Married	1,266 (51.3)	641 (52.4)	625 (51.0)	
	Divorced	258 (10.5)	119 (9.8)	139 (11.1)	
	Widowed	223 (9.0)	44 (3.6)	179 (14.2)	
Living with partner (yes), *n* (%)		1,501 (61.1)	759 (63.1)	742 (59.1)	0.047^c^
Living in a city (yes), *n* (%)		1,378 (55.7)	690 (56.8)	688 (54.6)	0.275^c^
Community size, *n* (%)	<5,000 residents	101 (4.1)	48 (4.0)	53 (4.2)	0.468^b^
	5,000–19,999 residents	227 (9.2)	119 (9.8)	108 (8.6)	
	20,000–99,999 residents	430 (17.4)	199 (16.4)	231 (18.3)	
	≥1,00,000 residents	1,718 (69.4)	849 (69.9)	869 (68.9)	

*Weighted random sample*.

a *Welch's t-test*.

b *Pearson's chi-squared test*.

c *Fisher‘s exact test; SD, standard deviation*.

### 6-Month Prevalence of Self-Reported Headache in Men and Women Across the Lifespan

In our sample, the weighted 6-month prevalence of self-reported headache amounted to 39.0%. 7.2% indicated to have received a diagnosis of migraine, 12.4% a diagnosis of TTH, and 23.4% had another diagnosis or had not received a diagnosis. 50.7% of the participants reporting headache had consulted a physician because of their headache, women (54.1%) more often than men (45.3%), $\chi_{\left(1,\;N\;=\;950\right)}^{2}$ = 6.9, *p* = 0.009.

Logistic regression analysis was employed to predict the probability of having headache in the last 6 months. Predictor variables were sex and age group (14–34 years, 35–54 years, 55–74 years, ≥75 years). The overall significance of the model was $\chi_{\left(4,\;N\;=\;2478\right)}^{2}$ = 3194.99, *p* < 0.001 (Likelihood-Ratio-Test). The analysis revealed both predictors remaining significant in the equation: sex (reference men) (*OR* = 2.22, 95%-*CI* 1.88–2.62, *p* < 0.001) and age groups (reference 14–34 years): 35–54 years (*OR* = 1.29, 95%-*CI* 1.05–1.60, *p* = 0.018), 55–74 years (*OR* = 0.97, 95%-*CI* 0.78–1.21, *p* = 0.800), ≥75 years (*OR* = 0.55, 95%-*CI* 0.40–0.76, *p* < 0.001). The odds ratio for sex indicated that women were 2.2 times more likely to have a headache than men. The age group variable was dummy coded using age group 14–34 years as the reference group. Participants aged 35–54 years were 1.29 times more likely to have a headache than participants aged 14–34 years. The odds of headache in age group 14–34 years were 1.82 times higher than for participants aged ≥75 years (inverted odds ratio for age group ≥75 years).

**Figure 1:**
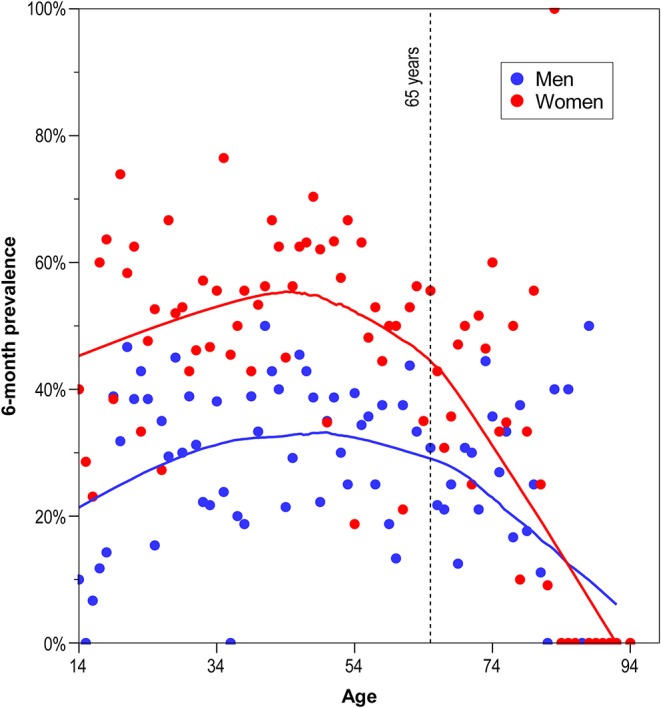
Weighted age-specific 6-month prevalence of self-reported headache, represented by scatterplot smoothing LOESS curves for men and women.

Univariate analyses indicated that the weighted prevalence of headache was higher in women compared to men in all headache categories: all headache types (male-to-female ratio 1:1.6), $\chi_{\left(1,\;N\;=\;2,477\right)}^{2}$ = 85.6, *p* < 0.001; migraine (male-to-female ratio 1:2.7), $\chi_{\left(1,\;N\;=\;2,477\right)}^{2}$ = 42.1, *p* < 0.001; TTH (male-to-female ratio 1:2), $\chi_{\left(1,\;N\;=\;2,477\right)}^{2}$ = 42.1, *p* < 0.001; other headaches (male-to-female ratio 1:1.3), $\chi_{\left(1,\;N\;=\;2,477\right)}^{2}$ = 15.2, *p* < 0.001. Prevalence rates of reported headache were 37.9% (14–34 years), 44.6% (35–54 years), 38.5% (55–74 years), and 26.9% (≥75 years). Participants aged 35–54 years were over-represented among headache participants (adjusted residual: 4.0), while participants aged ≥75 years less likely to have headache (adjusted residual: −4.4). In Figure 1, weighted age-specific 6-month prevalences are depicted, represented by scatterplot smoothing LOESS curves for men and women. Up to the age of around 45 years, the prevalence rose with age. After that age, prevalence declined with a steeper slope starting around 65 years (for more information see Supplement Table 1).

### Frequency of Headache in Men and Women Across the Lifespan

**Figure 2:**
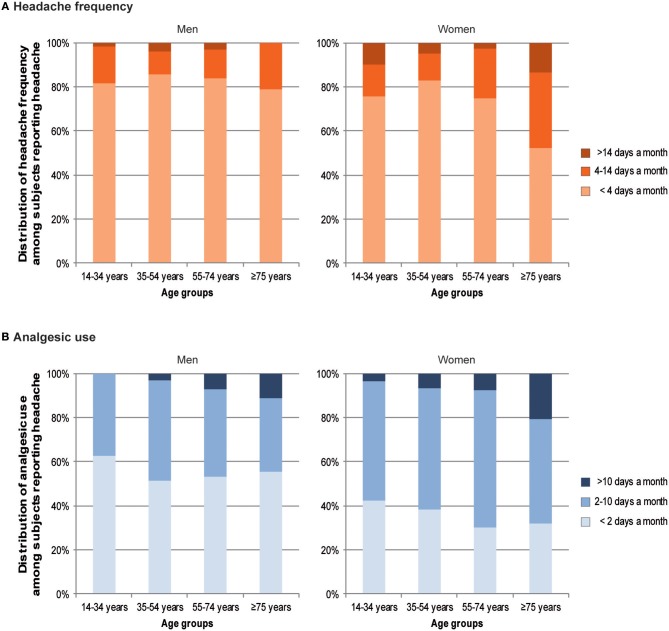
Headache frequency **(A)** and analgesic use **(B)** depending on age group.

Of the participants with headache, 79.5% reported headache on <4 days per month, 15.6% on 4–14 days per month and 4.9% on >14 days per month. We conducted a non-parametric two-way ANOVA to assess the effects of sex and age groups on the frequency of headache. The frequency of headache differed significantly for sex, $\chi_{\left(1,\;N\;=\;1,035\right)}^{2}$ = 20.73, *p* < 0.001, and age groups, $\chi_{\left(3,\;N\;=\;1,035\right)}^{2}$ = 7.20, *p* < 0.05. We found a significantly interaction between sex and age groups, $\chi_{\left(3,\;N\;=\;1,035\right)}^{2}$ = 13.92, *p* < 0.05. This interaction was due to the fact that women were under-represented among those with headache on <4 days per month (adjusted residual: −2.7). Given the hybrid type of interaction we conducted two separate non-parametric one-way ANOVAs for men and women. The frequency of headache did not differ significantly for age groups in men, $\chi_{\left(3,\;N\;=\;384\right)}^{2}$ = 1.45, *p* > 0.05, but in women, $\chi_{\left(3,\;N\;=\;651\right)}^{2}$ = 21.57, *p* < 0.001 (see Figure 2A). Women aged 35–54 years (adjusted residual: 3.0) were more likely and women aged ≥75 years (adjusted residual: −4.0) were less likely to have headache on <4 days per month. Additionally, women aged ≥75 years (adjusted residual: 3.1) were observed significantly more than expected among those with headache on 4–14 days per month. Detailed information on the frequency of headache regarding age group and sex is provided in the Supplement Table 1.

### Headache Impact in Men and Women Across the Lifespan

41.6% of the participants with headache reported no or little headache impact, 25.1% some impact, 12.2% substantial impact and 21.1% severe impact (HIT-6). We conducted a parametric two-way ANOVA to assess the effects of sex and age groups on the total score of HIT-6. The headache impact differed significantly for sex, *F*_(1;1, 005)_ = 36.34, *p* < 0.001. Women (mean: 52.78, *SD*: 8.66) reported a stronger impact than men (mean: 49.24, *SD*: 8.08). Age groups did not differ significantly, *F*_(3;1, 005)_ = 1.30, *p* = 0.274. Mean values for headache impact for the four age groups 14–34 years, 35–54 years, 55–74 years, ≥75 years were 51.29 (*SD*: 8.72), 51.52 (*SD*: 8.08), 51.14 (*SD*: 8.90), and 53.32 (*SD*: 10.51). There was no significant interaction effect between sex and age groups, *F*_(3;1, 005)_ = 0.98, *p* = 0.402.

### Frequency of Analgesic Use in Men and Women Across the Lifespan

Of the participants with headache, 43.3% reported taking analgesics on <2 days per month, 50.8% on 2–10 days per month and 5.9% on more than 10 days per month. A non-parametric two-way ANOVA was employed to assess the effects of sex and age groups on the frequency of analgesic use. The frequency of analgesic use differed statistically significant for sex, $\chi_{\left(1,\;N\;=\;999\right)}^{2}$ = 30.80, *p* < 0.001 and age groups $\chi_{\left(1,\;N\;=\;999\right)}^{2}$ = 9.52, *p* = 0.025. There was no significantly interaction between sex and age groups to the frequency of analgesic use, $\chi_{\left(3,\;N\;=\;999\right)}^{2}$ = 2.50, *p* > 0.05 (see Figure 2B). The significant association between sex and analgesic use was due to the smaller numbers of women (adjusted residual: −5.6) within those with <2 days per month and the increased numbers of women (adjusted residual: 4.6) among those with analgesic use on 2–10 days per month and >10 days per month (adjusted residual: 2.1). The significant association between age groups and analgesic use was attributable to the higher than expected number of participants aged ≥75 years (adjusted residual: 4.0) among those with analgesic use on >10 days per month (for more information see the Supplement Table 1).

### Frequency of Analgesic Use in Men and Women Across the Lifespan Depending on the Frequency of Headache

In the following, we tested whether there is a association between age group and analgesic use, respectively sex and analgesic use even if the participants belong to the same headache frequency category. We conducted parametric two-way ANOVAs for this purpose to assess the effects of sex and age groups on analgesic use (number of days per month) in three different headache frequency categories: <4 days per month, 4–14 days per month, and >14 days per month.

In the headache frequency category “ <4 days per month,” the analgesic use differed statistically significant for age groups *F*_(3;788)_ = 4.40, *p* = 0.004. The age group means showed a linear trend (*p* = 0.006). According to the Tukey-HSD, age group 14–34 years, mean: 1.75 (*SD*: 2.28), differed significantly from age group 55–74 years, mean: 3.22 (*SD*: 5.10) (−1.47, *p* = 0.002). Sex did not differ significantly, *F*_(1;788)_ = 2.44, *p* = 0.119. There was no significant interaction effect between sex and age groups, *F*_(3;788)_ = 0.35, *p* = 0.790.

In the headache frequency category “4–14 days per month,” the analgesic use differed neither in age groups, *F*_(3;153)_ = 1.08, *p* = 0.361, nor in sex, *F*_(1;153)_ = 1.30, *p* = 0.256. There was no significantly interaction effect between sex and age groups, *F*_(3;153)_ = 0.36, *p* = 0.790.

**Figure 3:**
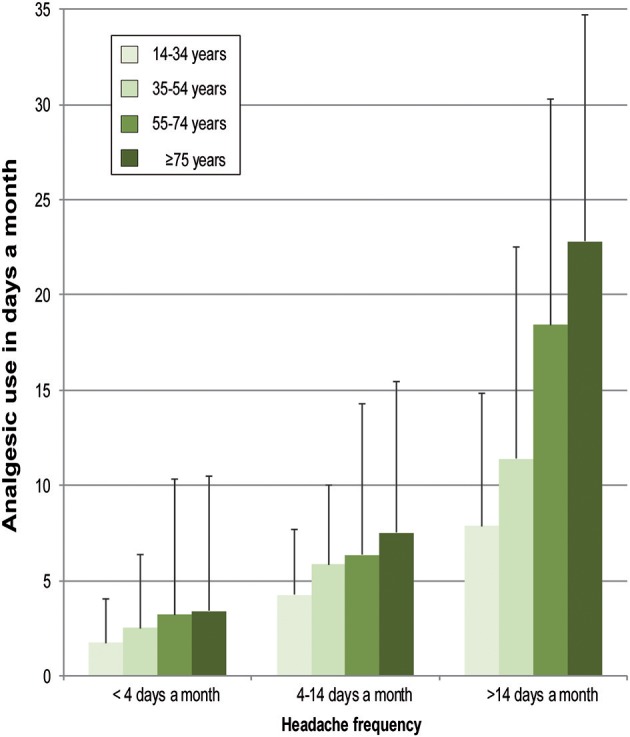
Frequency of analgesic use (days a month) depending on frequency of headache and age group.

In the headache frequency category “>14 days per month,” the analgesic use differed statistically significant for age groups, *F*_(3;43)_ = 4.09, *p* = 0.012. The age group means showed a linear trend (*p* = 0.003). According to the Tukey-HSD, age group 14–34 years, mean: 7.89 (*SD*: 6.96), differed significantly from age group ≥75 years, mean: 22.83 (*SD*: 11.84) (−14.94, *p* = 0.014). Sex did not differ significantly, *F*_(1;43)_ = 1.77, *p* = 0.191. There was no significant interaction effect between sex and age groups, *F*_(2;43)_ = 0.78, *p* = 0.465 (see Figure 3).

## Discussion

We analyzed the 6-month prevalence and characteristics of self-reported headache across the lifespan in a random general population sample in Germany aged 14–94 years.

In our study, the overall headache prevalence in the past 6 months in Germany, as obtained with a questionnaire, was 39%. This frequency is slightly lower than the mean prevalence in Europe (53%) reported by Stovner and Andree (4). They evaluated 49 studies with different timeframes of headache prevalence (a third of them contained lifetime prevalence data) and different methods of data collection (nearly a third of studies with a timeframe for headache ≤1 year were based on personal interviews). Both, lifetime prevalence of headache (compared to a timeframe for headache ≤1 year) and prevalence rates based on personal interviews (compared to questionnaires) result in higher figures. The prevalence rate in our German study is comparable to Norway (38%) (23), Estonia (41%) (24), and Italy (43%) (12). Furthermore, we found a 6-month prevalence of self-reported diagnosed migraine of 7.2% with a male-to-female ratio of 1:2.7. The mean prevalence of migraine in Europe is two times higher (14.7%) with a quite similar male-to-female ratio of 1:2.2 (14.7%) (4). The difference is most likely due to half of the men and women with migraine in Germany not knowing their headache diagnosis. This result supports findings of other studies (25, 26). The 6-month prevalence of self-reported diagnosed TTH in our study (12.4%) is approximately five times lower than the mean prevalence in Europe (62.6%) (4). The male-to female ratio in TTH of our sample (1:2) differs only slightly from that in other European studies (1:1.3). The proportions of women and men with TTH who do not know their diagnosis are similarly high. It is estimated that people with headache underreport TTH more often than migraine due to the lesser severity of symptoms and impact of TTH (27–30). 23.4% of all participants had another diagnosis or did not know their diagnosis, which corresponds to 60% of all those suffering from headache. This result is at glance surprising, as maximally 8–10% of all headache patients suffer from secondary headaches only, and just about 1–2% of the headache patients suffer from primary headaches other than migraine or TTH (31–36). Our conclusion is that about 35% of the population in Germany has primary headaches and that about 44% of the patients with primary headache are not aware of their specific diagnosis. This fits well with the result that 49% of the participants with headache had not seen a physician because of their headache (and consequently not received a diagnosis). However, diagnosing or classifying the individual headache allows for an appropriate treatment and management (37–41). The low consultation rate probably reflects the general opinion that headaches cannot be treated specifically, or that self-medication is sufficient.

We found that the headache prevalence was associated with sex and age group. Higher headache prevalence in women is well-documented in the literature (4, 16, 42, 43). Compared to the age group 14–34 years, participants aged 35–54 years are more and those aged ≥75 are less likely to have headache (inverted u-shape, see Figure 1). This likely reflects the known age-specific prevalence rates of migraine and TTH. Migraine reaches a peak during the fourth decade of life and remains constant during the fifth decade, TTH peaks between the ages of 30 and 39 years (44, 45). The lowest prevalence of TTH and migraine is at age 70 years and older (13, 42, 44, 46, 47).

Of the participants with headache, 80% reported headache on <4 days per month. Lipton et al. (29) reported a very similar proportion (79%) for participants with migraine in an American sample. Although in clinical practice, in addition to attack frequency, other criteria (e.g., duration of attack, impairment of quality of life) are considered when deciding on preventive treatment, we assume from an epidemiological perspective that about 80% of all participants with headache do not need preventive therapy (48). In our sample, of the participants with headache, 5% reported chronic headache (>14 days per month), which is about 2% of our total sample. Stovner et al. (49) estimated, based on data from 8 European countries, that about 4% of the adult population in Europe suffers from chronic headache. Data from Germany were not included. Our data suggest that the proportion among the population aged ≥14 in Germany is lower.

In the following we discuss the three most important results of our study:

First, we could show that in women, but not in men, the frequency of headache attacks differs between age groups. Women in old age (≥75 years) tend to have more headache days (per month) than younger women. This tendency is known for migraine patients (15, 50). However, it has not yet been analyzed in the literature whether this trend applies to both men and women. It is possible that the association between age and frequency of migraine attacks exists only in women. The cause of higher attack frequency in older patients is not known. It could be hypothesized that only patients with primarily severe migraine still have it in older age and that more patients with less severe migraine become headache-free in older age. In this case, the higher headache frequency in old age would be the result of a selection effect. Furthermore, the polypharmacy in older patients may exacerbate a pre-existing headache or trigger medication overuse headache (MOH), e.g., regular intake of analgesics for chronic arthritis pain (46, 51, 52).
Second, the impact of headache did not differ between age groups. We found no other study in which the impact of headache was analyzed across the lifespan. Given that among women the frequency of attacks increases with age, the finding of a stable headache impact appears paradoxical. Plausible would have been an increase in headache impact with age. We suppose that psychological adaptation processes, according to the theory of Rothermund and Brandstädter (53) about coping with deficits and losses in later life, play an important role in the subjective perception of the headache impact. It is possible that, based on the longer duration of the disease, headache sufferers in old age are better able to cope with their condition than younger ones. They may be more efficient able to generate affectively relieving cognitions.
Third, we could show that among participants with a headache frequency of >14 days per month those aged ≥75 years use analgesics on more days per month than younger participants. The reason is unclear. In our study, we used a categorical variable to measure headache frequency. We do not have data about the actual number of headache days per month. It is possible that older people with >14 headache days per month have a higher frequency of headache than younger people. In this case, the higher headache frequency could result in a higher burden of disease, which is compensated by a more frequent intake of analgesics. It is also conceivable, however, that older people with the same headache frequency as younger people may take more analgesics than younger ones. However, it could also be due to the intake of analgesics for treatment of concomitant (non-headache) painful conditions, which are more frequent with increasing age. Furthermore, the more frequent intake of analgesics can also be the reason for higher blood pressure in elderly persons (54). Studies indicate that roughly half of the individuals with headache on >14 days per month use analgesics daily or almost daily (55, 56). Our data suggest that the risk may be higher in old age.
Physicians should pay attention to the frequency of headache attacks and medication use in older people. The more frequent intake of analgesics might put older people at risk of developing medication overuse and subsequently MOH (57). Especially for older women who consult a physician for non-headache pain, taking the history should also include questions about self-medication to treat headache. General practitioners play a key role, as they are often consulted by older patients. They should be aware of the possibility of MOH and refer the patients to a specialist if necessary. Based on the assumption that the higher analgesic use of older subjects was mainly caused by headache, our data implies an unmet need of pharmacological management of headache in the elderly. Pharmacological headache trials should therefore not be limited to the age of 65, but should include also individuals aged 70–75 years or even older.

## Limitations

An important limitation is the fact that our headache type classification was based on participants' self-reports of prior headache diagnoses, necessarily underestimating migraine and TTH prevalence. It is possible that the category “other” includes many undiagnosed migraine and TTH patients. In this regard, our results on prevalence rates are less accurate than studies that reported prevalence based on ICHD III beta criteria. Furthermore, we cannot say whether the attacks and more frequent use of analgesics in old age are due to migraine or TTH, nor whether they are due to primary or secondary headache. We assume that the response to the question about the use of analgesics was influenced by an anchoring effect (58): the headache-related questions were preceded by an introductory sentence (“The following questions deal with headaches. Please answer as accurately as you can.”); the question about analgesic use was asked in the latter part of the headache-related questionnaire and followed immediately after three questions related directly to the treatment of headache. Nevertheless, we cannot rule out the possibility that the analgesics may have been taken because of other painful, not headache-related symptoms. Especially for chronic headache, strong associations to musculoskeletal symptoms and to fibromyalgia have been reported (59, 60). It is still unclear whether these relationships are moderated by age.

## Conclusion

In our representative German sample of participants aged ≥14 years and living in private households, about 39% suffer from headache, and many participants with headache do not know their diagnosis. We estimate this proportion at about 44%, based on the proportions of participants reporting migraine and TTH diagnoses compared to the known prevalences of these disorders. Headache prevalence peaks in the age group 35–54 years, the lowest prevalence was found in the age group 75 years and more. Women in old age have more headache days per month than younger women. Among participants with a headache frequency of >14 days per month, those aged ≥75 years use analgesics on more days per month than younger participants, putting them at risk for MOH. More research is needed to understand these specifics of headache in old age. As a first step toward an effective treatment of primary headaches in the general population, it also seems necessary to encourage people suffering from headache to contact a physician and receive a headache diagnosis.

## Data Availability Statement

The datasets generated for this study are available on request to the corresponding author.

## Author Contributions

TD, CG, TJ, PK, AS, and SF contributed conception of the study. BM and ÄG performed the statistical analysis. BM wrote the first draft of the manuscript. BM, TD, CG, TJ, PK, RR, SF, and AS reviewed and edited the manuscript. All authors approved the final version of the manuscript.

### Conflict of Interest

TD has received honoraria for consulting within the past 3 years from Novartis Pharma. CG has received honoraria for consulting and lectures within the past 3 years from Allergan Pharma, Bayer vital, Boehringer Ingelheim Pharma, Cerbotec, Desitin Arzneimittel, electroCore, Grünenthal, Hormosan Pharma, Lilly Germany, Novartis Pharma, Ratiopharm, Reckitt Benckiser, Sanofi Aventis, and TEVA. TJ has received honoraria for consulting and lectures from Allergan Pharma, Autonomic Technologies, Desitin Arzneimittel, Lilly Germany, Novartis Pharma, and TEVA. PK has received honoraria for consulting and lectures from Novartis Pharma and Shire. RR has received honoraria for consulting and lectures within the past 3 years from Allergan Pharma, Hormosan Pharma, Novartis Pharma, Lilly Germany, and TEVA. AS has received honoraria for consulting and educational lectures from Allergan Pharma, Bayer, Desitin Arzneimittel, electroCore, Lilly Germany, Novartis Pharma, Sanofi Aventis, and TEVA. SF has received honoraria for consulting and lectures within the past 3 years from Allergan Pharma, Astra Zeneca, Hormosan Pharma, Lilly Germany, Novartis Pharma, Sanofi Aventis, and TEVA. The remaining authors declare that the research was conducted in the absence of any commercial or financial relationships that could be construed as a potential conflict of interest.

## Supplementary Material

Supplement Table 1 Percentage of participants with headache reporting selected characteristics (in %, with 95% CI). Weighted random sample.

## Abbreviations

ICHD: International Classification of Headache Disorders
MOH: medication overuse headache
TTH: tension-type headache.
