# Supplementary material for: More Attacks and Analgesic Use in Old Age: Self-Reported Headache Across the Lifespan in a German Sample
Source: Front Neurol. 2019 Oct 17;10:1000. doi: 10.3389/fneur.2019.01000 (PMC6843053; doi:10.3389/fneur.2019.01000)
Supplement: Supplement Table 1 — Percentage of participants with headache reporting selected characteristics (in %, with 95% CI). Weighted random sample. [file Table_1.DOC]

*Supplement. Percentage of participants with headache reporting selected characteristics* (in %, with 95% CI). Weighted random sample

|  | | Total | | | Age group | | | | | | | | | | | |
| --- | --- | --- | --- | --- | --- | --- | --- | --- | --- | --- | --- | --- | --- | --- | --- | --- |
|  |  |  | | | 14-34 years | | | 35-54 years | | | 55-74 years | | | 75 years and more | | |
|  |  | All  n=967 | Men  n=362 | Wo-men  n=605 | All  n=256 | Men  n=103 | Wo-men  n=153 | All  n=362 | Men  n=131 | Wo-men  n=231 | All  n=274 | Men  n=98 | Wo-men  n=176 | All  n=74 | Men  n=30 | Wo-men  n=44 |
| Frequency of headache | < 4 days a month | 79.5 (77.0, 82.1). | 84.3 (80.6, 88.0) | 76.7 (73.3, 80.1) | 78.1 (73.0, 84.2) | 81.6 (74.1, 89.1) | 75.8 (69.0, 82.6) | 84.0 (80.2, 87.8) | 85.5  (79.5, 91.5) | 83.1 (78.3, 87.9) | 78.1 (73.2, 83.0) | 83.7 (76.4, 91.0) | 75.0 (68.6, 81.4) | 67.6 (56.9, 78.3) | 90.0 (79.3, 100.0) | 52.3 (37.5, 67.1) |
|  | 4-14 days a month | 15.6 (13.3, 17.9) | 13.0 (9.5, 16.5) | 17.2 (14.2, 20.2) | 15.2 (10.8, 19.6) | 16.5 (9.3, 23.7) | 14.4 (8.8, 20.0) | 11.6 (8.3, 14.9) | 10.7 (5.4, 16.0) | 12.1 (7.9, 16.3) | 19.0 (14.4, 23.7) | 13.3 (6.6, 20.0) | 22.2 (16.1, 28.3) | 24.3 (14.5, 34.1) | 10.0  (0.0, 20.7) | 34.1 (20.1, 48.1) |
|  | >14 days a month | 4.9  (17.7, 22.3) | 2.8 (1.1, 4.5) | 6.1 (4.2, 8.0) | 6.6 (3.6, 9.6) | 1.9 (0.0 4.6) | 9.8 (5.1, 14.5) | 4.4 (2.3, 6.5) | 3.8 (0.5, 7.1) | 4.8 (2.0, 7.6) | 2.9 (0.9, 4.9) | 3.1 (0.0, 6.5) | 2.8 (0.4, 5.2) | 8.1 (1.9, 14.3) | 0 | 13.6 (3.5, 23.7) |
| Headache Impact (HIT-6)^c^ | None/ mild | 66.7 (63.8, 69.7) | 76.5 (72.0, 81.0) | 61.0 (57.1, 64.9) | 68.7 (63.1, 74.4) | 81.4 (73.9, 88.9) | 60.0 (52.2, 67.8) | 66.8 (61.9, 71.7) | 73.2 (65.6, 80.8) | 63.3 (57.1, 69.5) | 66.9  (61.3, 72.5) | 77.4 (69.1, 85.7) | 61.3 (54.1, 68.5) | 58.2 (47.0, 69.4) | 69.2 (52.7, 85.7) | 51.2 (37.5, 67.1) |
|  | Moderate/ severe | 33.3 (30.3, 36.3) | 23.5 (20.1, 26.9) | 39.0 (35.1, 42.9) | 31.3 (25.6, 37.0) | 18.6 (11.1, 26.1) | 40.0 (32.2, 47.8) | 33.2 (28.3, 38.1) | 26.8 (19.2, 34.4) | 36.7 (30.5, 42.9) | 33.1 (27.5, 38.7) | 22.6 (14.3, 30.9) | 38.7 (31.5, 45.9) | 41.8 (30.6, 53.0) | 30.8  (14.3, 47.3) | 48.8 (34.0, 63.6) |
| Frequency of analgesic use | < 2 days a month | 43.3 (40.2, 46.4) | 55.2 (50.1, 60.3) | 36.4 (32.6, 40.2) | 50.2 (44.1, 56.3) | 62.5  (53.2, 71.9) | 42.2 (34.4, 50.0) | 43.0 (37.9, 48.1) | 51.2 (42.6, 59.8) | 38.4 (32.1, 44.7) | 38.4 (32.6, 44.2) | 53.1 (43.2, 63.0) | 30.2 (23.4, 37.0) | 40.8 (29.6, 52.0) | 55.6 (37.8, 73.4) | 31.8 (18.0, 45.6) |
|  | 2-10 days a month | 50.8 (88.1, 93.9) | 41.0 (35.9, 46.1) | 56.5 (52.5, 60.5) | 47.7 (41.6, 53.8) | 37.5 (28.2, 46.9) | 54.4 (46.5, 62.3) | 51.6 (46.5, 56.8) | 45.6 (37.1, 54.1) | 54.9 (48.5, 61.3) | 54.1 (48.2, 60.0) | 39.6 (29.9, 49.3) | 62.2 (55.1, 69.4) | 42.3  (31.1, 53.6) | 33.3 (16.4, 50.2) | 47.7 (32.9, 62.5) |
|  | >10 days a month | 5.9 (4.4, 7.4) | 3.8 (1.8, 5.8) | 7.1 (5.1, 9.1) | 2.1 (0.0, 3.9) | 0 | 3.4 (0.0, 6.3) | 5.4 (3.1, 7.7) | 3.2 (0.0, 6.2) | 6.7 (3.5 9.9) | 7.5 (4.4, 10.6) | 7.3 (2.1, 12.5) | 7.6 (3.7, 11.5) | 16.9 (8.4, 25.5) | 11.1 (0.0, 22.4) | 20.5 (8.6, 32.4) |

HIT-6, Headache Impact Test-6; CI, confidence interval.
